# Psychometric Evaluation of the Persian Version of Barkley Adult Attention Deficit/Hyperactivity Disorder Screening Tool among the Elderly

**DOI:** 10.1155/2017/9109783

**Published:** 2017-11-01

**Authors:** Mostafa Sadeghi, Homayoun Sadeghi-Bazargani, Shahrokh Amiri

**Affiliations:** ^1^Faculty of Health, Department of Biostatistics & Epidemiology, Tabriz University of Medical Science, Tabriz, Iran; ^2^Road Traffic Injury Research Center, Statistics & Epidemiology Department, Tabriz University of Medical Sciences, Tabriz, Iran; ^3^Research Center of Psychiatry and Behavioral Sciences, Tabriz University of Medical Sciences, Tabriz, Iran

## Abstract

**Background:**

The Barkley Adult Attention Deficit/Hyperactivity Disorder (ADHD) Rating Scale-IV (BAARS-IV) was developed, and it demonstrated good psychometric properties. The BAARS-IV includes 27 questions on the symptoms of adult ADHD. The purpose of the present study is to investigate the psychometric testing of the Persian version of BAARS-IV among the elderlies in Tabriz City.

**Method:**

This cross-sectional study was conducted in Tabriz City—in the west of Iran—in 2015 via enrolling of 121 old-aged people. We did the process of translation and adaptation of BAARS-IV and examined its concurrent validity, internal consistency, and test-retest reliability.

**Result:**

The BAARS-IV demonstrated good internal consistency and test-retest reliability. Correlations between the BAARS-IV and the CAARS-S: SV were high and evidence supporting concurrent validity was revealed. Cronbach's alpha for the overall scale and subscales stood at 0.89, 0.81, 0.66, 0.56, and 0.82, respectively.

**Conclusion:**

The Persian BAARS-IV showed acceptable reliability and validity. BAARS-IV was determined to be composed of internally consistent and psychometrically sound items.

## 1. Introduction

Attention Deficit/Hyperactivity Disorder (ADHD) is among the most common neurobehavioral disorders seen in the people referring to the children-adult psychiatric clinics [1]. This disorder includes people having issues in their home, school, or social life manifesting symptoms of inattention, hyperactivity, and impulsivity [2]. Despite an earlier belief that ADHD only persists in children, recent studies show that ADHD also continues in adulthood [3].

Thus far, most studies in adulthood concern young or middle-aged adults, but little literature is present with regard to the elderly [4]. Elderlies with ADHD show symptoms of distractibility and disorganization at home and in their work-related environment [5]. Their problems of inattention manifest in disorganization, forgetfulness, unreliability, and difficulty in planning and/or in completing tasks [5]. Also, difficulties in relationship with peers and family members appeared through marital discords and paradoxical relationship with friends [5].

A study in Netherlands reported prevalence of syndromic ADHD in elderlies as 2.8 and prevalence of symptomatic ADHD at 4.2, by using DSM-IV criteria. The prevalence was higher in elderlies aged between 60 and 70 in comparison with the ones aged 71 to 94 [4]. The study found that 63% of elderlies with ADHD also suffered from psychiatric disorders, came from lower educational backgrounds, had poor job performance, and were socially more isolated. Older ones had more problems in productivity and their outlook in life [6]. However, little is known about ADHD among the elderly, and because ADHD in elderlies is a relatively new mental health disorder, a valid screening tool is required [7]. Without doubt, a standard screening tool would be required to ensure the possibility of diagnosing this disorder in a wide range of clinical and researching studies [7].

*Choice for the BAARS-IV*. The Barkley Adult ADHD Rating Scale-IV (BAARS-IV) is meant to replace the current symptoms scale [8]. The self-report version of current symptoms scale includes 30 items. 27 of the 30 items are rated on a 4-point Likert scale: (1) never or rarely, (2) sometimes, (3) often, and (4) very often. Each scale of current symptom has three extra questions. The New BAARS-IV has additional nine items for evaluating the symptoms of Sluggish Cognitive Tempo (SCT) [9, 10]. SCT includes symptoms such as daydreaming, staring, mental fogginess, confusion, hypoactivity, sluggishness, slow movement, lethargy, apathy, and sleepiness [9, 11–14]. SCT symptoms show a stronger relationship with internal isolation and social withdrawal [10, 13, 14]. The internal consistency data for this version of the tool was rated from good to excellent [15]. Moreover, the sensitivity and specificity of this version were highly acceptable, and therefore it was recommended in clinical use for diagnosing ADHD among adults [16–18]. One study of the Barkley Adult ADHD Rating Scale-IV was based on the DSM-IV-TR criteria having a positive predictive power ranging from 0.78 to 0.91 across the subscales and a negative predictive power ranging from 0.84 to 0.98 [16].

In fact, Barkley Adult ADHD Rating Scale-IV was adequately valid and predictive of disorder. This scale is highly recommended as first step screening in clinical evaluation of ADHD among adults. Thus considering all features of this tool and the absence of an accurate tool for diagnosing such disorders in elderlies, this study was carried out to psychometrically test the Persian version of BAARS-IV among elderlies in Tabriz-Iran.

## 2. Methods

### 2.1. Study Population

This cross-sectional study was conducted in Tabriz City, the west of Iran, in 2015 enrolling 121 old-aged people (over 60 years old). In order to recruit these participants, we selected Golgasht district of Tabriz City that has a population with a range of low to high socioeconomic status inhabiting it. The sampling method used was cluster sampling in which we chose three clusters and the people were selected according to convenience sampling. Also to consider psychiatric disorders, medical records of persons were obtained. In case of facing any subject suspected of having psychiatric disorder, we considered psychiatric visit using the Structured Clinical Interview (SCID) on the basis of DSM-IV-TR criteria [19], but no such cases were encountered.

### 2.2. The Scale Development Process

**Figure 1:**
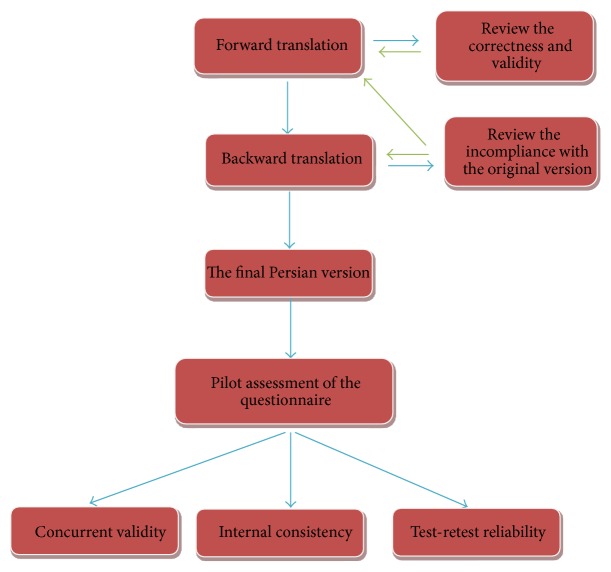
The development process and evaluation of the Persian version of BAARS-IV.

The process for development and assessment of the tool is shown in Figure 1. Psychometric evaluation of the Persian version of BAARS-IV involved translation (forward and backward), concurrent validity, internal consistency, and test-retest reliability.

In translation phase, we translated the tool based on the standards of WHO's translation and adaptation protocol. A person fluent in English (in the native tongue) and familiar with the concepts translated the questionnaire into Persian. After reviewing the correctness and validity of the translation and editing it according to the Persian and English language professor's comments, necessary changes with formal and conceptual compliance (with priority in conceptual compliance) were applied by a panel of experts (including eight psychiatrists and an epidemiologist). The Persian version of the questionnaire, on the other hand, was first translated by an Iranian person living abroad—without seeing the original English items of the questionnaire. After that the panel of experts accomplished the process of evaluating and conceptual compliance of the retranslated sentences with the original ones in the English version, and eventually the questionnaire was finalized.

### 2.3. Assessment of Concurrent Validity

A Persian version of Conner's Adult ADHD Rating Scales (self-report version) which is one of the most substantial and practical tools used for diagnosing and screening adult ADHD [20] was administered. The concurrent validity was assessed via ICCs and also Kendall's Tau and Bland-Altman Plot.

### 2.4. Assessment of Internal Consistency Reliability

In order to determine the internal consistency reliability of questions, Cronbach's alpha coefficient was used for total scale and each subscale separately. Plus, to ascertain test-retest reliability, the questionnaires were distributed simultaneously among 60 older adults in two weeks and then the process was assessed by ICC.

A value of above 0.7 for Cronbach's alpha and one higher than 0.5 for ICC were considered as acceptable reliability. The value of  .05 was considered as the statistical significance level for all tests.

STATA (version 13) statistical software packages were used for the analysis of data.

### 2.5. Ethical Issues

The study protocol was approved by the regional committee of ethics in Tabriz University of Medical Science under the Ethical Registration Number 660. Verbal informed consent was obtained from all the participants.

In order to receive the original questionnaire, we communicated with Dr. Barkley. He gave us the questionnaire with his own permission after we explained the methodology and aims of our study.

## 3. Results

One hundred and twenty-one aged people were enrolled in this study. 67 persons (55.37%) were male and 54 (44.63%) of them were female. The mean age of the participants was 68.15 ± 7.99 years.

### 3.1. Evaluating the Quality of Translation and Preparing the Final Persian Version of BAARS-IV

**Figure 2:**
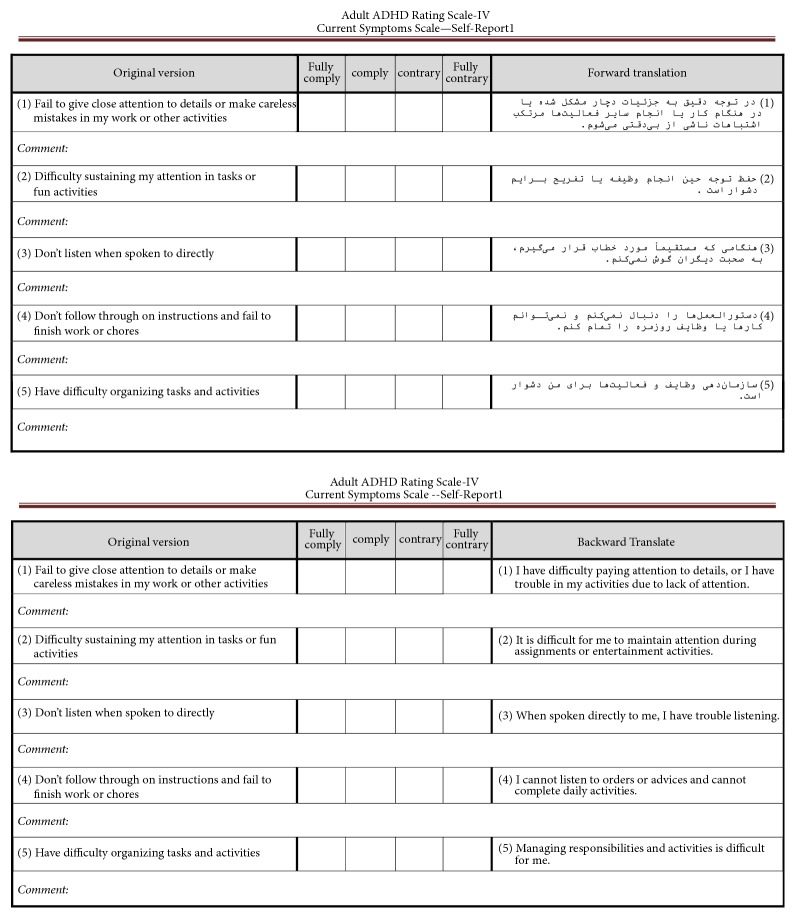
A sample section on the process of accessing compatibility forward and backward translation with the original version.

After the forward and backward translation process, we shared it with the panel of experts and collected their comments; then the necessary modifications were made using these comments through simplification or replacement of words or phrases as required in the meaning (Figure 2).

### 3.2. Concurrent Validity

ICCs and Kendall's tau-b correlations between the BAARS-IV and CAARS-S: SV were calculated at 0.88 and 0.69, respectively.

Table 1 illustrates ICC's and Kendall's tau-b correlations between the BAARS-IV and CAARS-S: SV scale.

**Figure 3:**
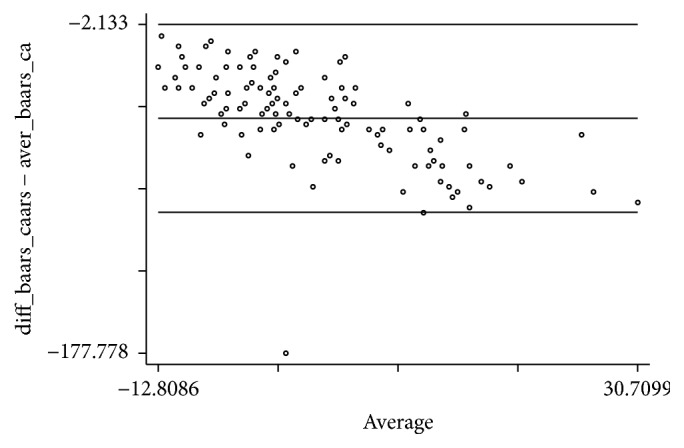
A Bland-Altman plot for agreement between the BAARS-IV and CAARS-S: SV scale.

Figure 3 illustrates Bland-Altman plot for agreement between the BAARS-IV and the CAARS-S: SV scale.

### 3.3. Internal Consistency Reliability

Table 2 shows the internal consistency reliability. The questionnaire BAARS-IV includes 4 factors with Cronbach's alpha equal to 0.81, 0.66, 0.56, and 0.82 for ADHD Inattention, ADHD Hyperactivity, ADHD Impulsivity, and Sluggish Cognitive Tempo (SCT), respectively.

### 3.4. Test-Retest Reliability

Table 2 shows the test-retest reliability situation for each factor separately. ICC is higher than 0.5 for all factors, which is acceptable.

## 4. Discussion

It is a given fact that adult ADHD draws extensive scientific interest in the West, yet it remains less known in Iran. As we mentioned, Persian literature on adult ADHD is practically nonexistent and its prevalence is unknown since all local studies focus on ADHD in child population. Despite the fact that there is a general agreement recognizing ADHD as having a reliable diagnosis in child population through the official diagnostic criteria, there is less agreement on it as a valid diagnostic tool in elderly population [21].

Unfortunately, no efforts have been taken to establish a reliable and valid tool to assess ADHD symptoms in the elderly despite the growing need to render services to this expanding population. Therefore, the aim of this study is to provide a Persian version of BAARS-IV, which is well-established as a rating scale of ADHD symptoms and developed in USA [15], considering that the examination of its applicability in Iran would be a starting point to provide a valid and stable tool for elderly population.

Basically, tests for mental health are designed in one country and used by others, by taking into account the existing cross-national and cross-cultural differences. Translating an instrument from the source to the target language is a rather complicated matter, the goal of which is to stay as close as possible to the semantic, conceptual, and technical aspects of the original found in the different versions of the instruments [22]. The BAARS-IV was translated into Persian via a tedious method. Needless to say, few conceptual differences were found that made the process of translation a little more difficult. However, on the whole, the questions in the Persian format are both acceptable and comprehensible.

Psychometric assessment of Persian version of BAARS-IV proves that it is a valid and stable tool for screening ADHD in elderlies. Classic psychometric analysis confirmed the scale capability for sound measurement with adequate psychometric properties.

Internal consistency was examined using Cronbach's alpha for the BAARS-IV total and subscale scores: in particular, alpha coefficients of the Current ADHD Inattention, Hyperactivity, and Impulsivity subscales (range of *α*'s = .56–.82) and the Current ADHD total score (*α* = .89). In the original tool, Current ADHD Inattention = .902, Current ADHD Hyperactivity = .776, Current ADHD Impulsivity = .807, and Current ADHD total score = .914 [15]. The two rang Hyperactivity and Impulsivity subscales were low in our research in comparison with the original version which could be because the original version is not specialized for elderlies. Therefore, it has low sensitivity and specificity which can have effect on reliability.

The stability of test/retest was moderate after two weeks (0.69). Although ADHD is a chronic disorder and persist over time. The moderate test-retest reliability may have been caused by slight variability in the self-perceived symptoms on the two measurements. It could also be that the questions are not specific enough for older adults to recognize the symptoms or that they experience other problems in their daily life caused by ADHD.

We used Conner's Adult ADHD Rating Scales for concurrent validity analysis which is a similar tool for assessing adult ADHD. A positive connection between BAARS-IV and CAARS-S: SV was observed in all subscales.

## 5. Limitations

The first limitation was that the tool (BAARS-IV) was not only specialized for elderlies. Another was that the tool used for concurrent validity analysis was in the form of a self-report questionnaire which may inflate the connection between research variables. More valid researches need to be done by clinical interviews as a golden standard and finally BAARS-IV screening must be undergone more times.

## 6. Conclusion

The Persian BAARS-IV showed good reliability and satisfactory validity. This research suggests that the BAARS-IV appears to be a promising measure of older adult ADHD. The BAARS-IV was determined to be composed of internally consistent and psychometrically sound items.

Considering all features of this tool and absence of an accurate tool for diagnosing ADHD in elderlies, it has been recommended to create a specialized tool with high sensitivity and high specificity for ADHD in elderlies in future researches.

## Figures and Tables

**Table 1:** Kendall's tau-b and ICC^*∗*^ correlations between the BAARS-IV and CAARS-S: SV.

	Correlation coefficient	Significance (two-tailed)
ICC	0.88	0.000
Kendall's tau-b	0.69	0.000

*Note*. ^*∗*^Intraclass correlation.

**Table 2:** Situation of the internal consistency and test-retest reliability for each domain.

Domain	Number of questions	Cronbach's alpha	Mean of interitem correlation	Internal consistency reliability	ICC^*∗*^ (95% confidence interval)	Test-retest reliability
ADHD Inattention	9	0.81	0.35	Suitable	0.62 (0.37–0.77)	Medium
ADHD Hyperactivity	5	0.66	0.38	Medium	0.51 (0.19–0.71)	Medium
ADHD Impulsivity	4	0.56	0.27	Medium	0.82 (0.70–89)	Suitable
Sluggish Cognitive Tempo	9	0.82	0.35	Suitable	0.73 (0.55–0.84)	Suitable

*Note*. Total Cronbach's alpha and total Intraclass correlation were 0.89 and 0.69, respectively/^*∗*^Intraclass correlation.
